# *Idh2* Deficiency Exacerbates Acrolein-Induced Lung Injury through Mitochondrial Redox Environment Deterioration

**DOI:** 10.1155/2017/1595103

**Published:** 2017-12-31

**Authors:** Jung Hyun Park, Hyeong Jun Ku, Jin Hyup Lee, Jeen-Woo Park

**Affiliations:** ^1^Department of Food and Biotechnology, Korea University, Sejong, Republic of Korea; ^2^School of Life Sciences and Biotechnology, BK21 Plus KNU Creative BioResearch Group, College of Natural Sciences, Kyungpook National University, Taegu, Republic of Korea

## Abstract

Acrolein is known to be involved in acute lung injury and other pulmonary diseases. A number of studies have suggested that acrolein-induced toxic effects are associated with depletion of antioxidants, such as reduced glutathione and protein thiols, and production of reactive oxygen species. Mitochondrial NADP^+^-dependent isocitrate dehydrogenase (*idh2*) regulates mitochondrial redox balance and reduces oxidative stress-induced cell injury via generation of NADPH. Therefore, we evaluated the role of *idh2* in acrolein-induced lung injury using *idh2* short hairpin RNA- (shRNA-) transfected Lewis lung carcinoma (LLC) cells and *idh2*-deficient (*idh2^−/−^*) mice. Downregulation of *idh2* expression increased susceptibility to acrolein via induction of apoptotic cell death due to elevated mitochondrial oxidative stress. *Idh2* deficiency also promoted acrolein-induced lung injury in *idh2* knockout mice through the disruption of mitochondrial redox status. In addition, acrolein-induced toxicity in *idh2* shRNA-transfected LLC cells and in *idh2* knockout mice was ameliorated by the antioxidant, N-acetylcysteine, through attenuation of oxidative stress resulting from *idh2* deficiency. In conclusion, *idh2* deficiency leads to mitochondrial redox environment deterioration, which causes acrolein-mediated apoptosis of LLC cells and acrolein-induced lung injury in *idh2^−/−^* mice. The present study supports the central role of *idh2* deficiency in inducing oxidative stress resulting from acrolein-induced disruption of mitochondrial redox status in the lung.

## 1. Introduction

Acrolein is a ubiquitous environmental pollutant that arises from cigarette smoke, incomplete combustion of plastic materials, and pyrolyzed animal and vegetable; it is also endogenously produced during inflammation or oxidation of unsaturated lipids [1]. Acrolein inhalation results in the induction of gene regulation, inflammation, and lung cell apoptosis and necrosis [1]. It has been reported that exposure to acrolein leads to acute lung injury, disruption of alveolar capillary barrier integrity, pulmonary edema, and chronic obstructive pulmonary disease [2, 3].

It has been reported that acrolein causes oxidative stress by inducing, directly or indirectly, the production of excessive reactive oxygen species (ROS) that promote cellular apoptosis [4, 5]. ROS play a particularly important role in acrolein-induced cellular damage because acrolein is one of the most reactive *α*,*β*-unsaturated aldehyde products of lipid peroxidation [6, 7]. As an *α*,*β*-unsaturated aldehyde, acrolein contains a highly reactive carbonyl group and an electrophilic *α*-carbon that is highly reactive to cellular nucleophiles, such as proteins, DNA, and RNA [7]. Acrolein readily targets and reacts with the sulfhydryl group of cysteines to form thioether adducts via a Michael addition mechanism [8]. Depletion of cellular reduced glutathione (GSH) by the formation of GS-acrolein conjugates results in increased oxidative stress [9–11]. Acrolein can also deplete protein thiols, such as thioredoxin and glutaredoxin, which are important antioxidant proteins [12, 13].

Acrolein has also been reported as a mitochondrial toxicant, suggesting that it participates in mitochondrial dysfunction [14]. The mitochondria are one of the most important organelles involved in the production of ROS because the respiratory chain in the mitochondria is one of major sources of ROS production [15]. Additionally, these are also the main targets of ROS and oxidative stress-induced damage, as observed in various pathological states [16, 17]. Under normal physiological conditions, cell viability and function are critically dependent on the continued balance between mitochondrial ROS formation and removal [18]. ROS can be eliminated by antioxidant enzymes, such as superoxide dismutases, catalase, glutathione peroxidase, and peroxiredoxins (Prxs) [19]. In this regard, knockdown or inhibition of antioxidant enzymes can disrupt redox balance and exacerbate ROS-induced cell death. To maintain GSH-dependent mitochondrial antioxidant defense systems, the availability of the mitochondrial NADPH pool is critical [20]. In addition, the mitochondrial thioredoxin system, which includes thioredoxin 2 (TRX2) and thioredoxin reductase 2 (TRXR2), provides a disulfide reductase activity that is required for maintaining mitochondrial proteins in their reduced state. The mitochondrial thioredoxin system can interact with Prx3, exclusively detected in the mitochondria. Reduced TRX2 is regenerated by TRXR2 at the expense of NADPH [18]. The major enzyme to generate mitochondrial NADPH is the mitochondrial isoenzyme of NADP^+^-dependent isocitrate dehydrogenase (IDH2) [21, 22]. Thus, suppression of IDH2 activity may induce an imbalance of the mitochondrial redox state that subsequently increases the vulnerability of lung cells and tissues to acrolein-based modulation of the redox status.

The present study demonstrates that acrolein exposure promotes the inhibition of *idh2* expression, lowers the cell reduction potential, and increases ROS levels. Suppression of *idh2* expression led to disruption of mitochondrial redox status, induction of apoptosis, and acute injury in the lung of *idh2*-deficient (*idh2^−/−^*) mice and *idh2* short hairpin RNA- (shRNA-) transfected cells. These results suggest that attenuation or deficiency *of idh2* leads to increased mitochondrial ROS levels that causes acrolein-mediated apoptosis of Lewis lung carcinoma (LLC) cells and acrolein-induced lung injury in *idh2^−/−^* mice. The findings of the present study support a significant role for increased ROS resulting from disruption of mitochondrial antioxidant defense via suppression of IDH2 expression in acrolein-induced acute lung injury.

## 2. Materials and Methods

### 2.1. Materials

Propidium iodide (PI), 5,5′,-dithio-bis(2-nitrobenzoic acid), 3-(4,5-dimethylthiazol-2-yl)-2,5-di-phenyltetrazolium bromide (MTT), anti-rabbit IgG tetramethylrhodamine isothiocyanate- (TRITC-) conjugated secondary antibody, xylenol orange, N-acetyl-L-cysteine (NAC), and rhodamine 123 (Rh-123) were purchased from Sigma-Aldrich (St. Louis, MO), while 2′,7′-dichloro-fluorescin diacetate (DCFH-DA), diphenyl-1-pyrenylphosphine (DPPP), 3′-tetraethylbenzimidazolocarbocyanine iodide (JC-1), 5-chloromethylfluorescein diacetate (CMFDA), and MitoSox were purchased from Invitrogen (Eugene, OR). The antibodies used in this study were as follows: *β*-actin and cellular tumor antigen p53 (Santa Cruz Biotechnology, Santa Cruz, CA); p-JNK, cleaved-PARP, cleaved caspase-3, cleaved caspase-9, and horseradish peroxidase- (HRP-) conjugated secondary antibodies (Cell Signaling Technology, Beverly, MA); apoptosis regulator BAX (Calbiochem, San Diego, CA); acrolein adducts (Abcam, Cambridge, MA); and oxidized Prx (Prx-SO_3_) (Abfrontier, Seoul, Korea). A peptide containing the 16 N-terminal amino acids of mouse IDH2 (ADKRIKVAKPVVEMPG) was used to prepare polyclonal anti-IDH2 antibodies.

### 2.2. Cell Culture

The LLC cell line was purchased from the Japanese Collection of Research Bioresources Cell Bank (JCRB Cell Bank, Osaka, Japan). Cells were cultured using Dulbecco's modified Eagle's medium supplemented with 10% fetal calf serum and 1% penicillin/streptomycin in a humidified atmosphere at 37°C and 5% CO_2_. After 48 h, the plates were washed with phosphate-buffered saline (PBS) and incubated with new medium containing acrolein, while the same quantity of PBS was used as the control. Cells were cultivated in a humidified atmosphere at 37°C and 5% CO_2_ and harvested after acrolein exposure. The MTT assay was used to determine cell cytotoxicity as previously described [23].

### 2.3. *Idh2* shRNA Knockdown

*Idh2* shRNA and nontarget shRNA MISSION® lentiviral transduction particles were purchased from Sigma-Aldrich. LLC cells were transduced with a final concentration of 8 *μ*g/mL hexadimethrine bromide, according to the manufacturer's protocol. Transduced cells were selected as single colonies in a medium containing 5 *μ*g/mL puromycin (Clontech, Mountain View, CA) and maintained in a medium containing 1 *μ*g/mL puromycin.

### 2.4. RNA Isolation and Reverse Transcription Polymerase Chain Reaction (RT-PCR)

The RNA was extracted from LLC cells using an RNeasy kit (Qiagen, Hilden, Germany) in accordance with the manufacturer's instructions. RNA was reverse transcribed to cDNA using a first-strand cDNA synthesis kit (Invitrogen), according to the manufacturer's protocol. cDNAs were PCR-amplified. Sequences of the primers used were as follows: *β-actin*, forward: 5′-TCTACAATGAGCTGCGTGTG-3′, reverse: 5′-ATCTCCTTCTGCATCCT-GTC-3′ and *idh2*, forward: 5′-ATCAAGGAGAAGC-TCATCCTGC-3′, reverse: 5′-TCTGTGGCCTTGTACTGGTCG-3′. *β*-Actin was used as an internal control. The amplified DNA products were resolved on a 1% agarose gel and stained with ethidium bromide.

### 2.5. Flow Cytometric Analysis

Cells were collected at 2000*g* for 5 min and washed twice with cold PBS. Annexin V and PI staining were performed with the Alexa Fluor 488 Annexin V/Dead Cell Apoptosis Kit, according to the manufacturer's protocol. The stained cells were analyzed by flow cytometry (BD Biosciences, Franklin Lakes, NJ).

### 2.6. Assessment of Cellular Redox Status

Intracellular peroxide levels were measured using the ferric-sensitive dye xylenol orange and DCFH-DA as previously described [21]. Protein oxidation was assessed by immunoblot analysis using anti-Prx-SO_3_ antibody. Intracellular GSH levels were measured using a GSH-sensitive fluorescent dye, CMFDA. Cells were stained with 5 *μ*M CMFDA for 30 min at 37°C.

### 2.7. Cellular Oxidative Damage

Thiobarbituric acid-reactive substances (TBARS) were used for measurement of lipid peroxidation. Cell extracts were mixed with 1 mL TBA solution [0.375% thiobarbituric acid in 0.25 N HCl containing 15% (*w*/*w*) trichloroacetic acid] [21]. Lipid peroxidation was also detected by using a fluorescent DPPP probe [24]. The levels of 8-hydroxy-2′-deoxyguanosine (8-OH-dG) in LLC cells were measured with a fluorescent binding assay as described previously [25]. Cells were fixed and permeabilized with ice-cold methanol for 15 min. DNA damage was visualized with avidin-conjugated TRITC (1 : 200 dilution) using a fluorescence microscope. The comet assay was also performed using the Comet Assay Kit (Cell Biolabs Inc., San Diego, CA). Cell extracts were washed with cold PBS and centrifuged. The cell pellet was mixed with Comet Agarose at 1 : 10 ratio (*v*/*v*) and pipetted onto the Comet assay slide. Slides were dried at 4°C in the dark for 15 min and incubated in chilled lysis solution at 4°C in the dark for another 15 min. After washing with TBE buffer (50 mM Tris, 50 mM boric acid, and 0.2 mM EDTA), the samples were subjected to electrophoresis and stained with Vista Green DNA Dye. Images were obtained with a microscope. The percentage of tail DNA of the cells in each slide was measured and quantified.

### 2.8. Measurement of Mitochondrial Redox Status and Damage

Healthy mitochondrial membrane potentials were detected using the fluorescent probe JC-1 (Invitrogen). Cells were incubated at 37°C with 5 *μ*M JC-1 for 30 min. The ratio of the intensity of green/red fluorescence is directly proportional to the mitochondrial membrane potential [26]. The mitochondrial membrane permeability transition (MPT) was visualized using the fluorescent probe Rh-123, and mitochondrial ROS level was measured using MitoSox. Cells were grown in 100 mm plates containing a slide glass coated with poly-L-lysine and treated with acrolein or PBS and were treated for 30 min with 5 *μ*M each of Rh-123 and MitoSox. Slides with cells on top were washed in warm PBS and covered with a glass cover slip. Rh-123 fluorescence (excitation/emission: 500/536 nm) and oxidized MitoSox red fluorescence (excitation/emission: 510/580 nm) were imaged on a Zeiss Axiovert 200 inverted microscope and a Zeiss LSM700 confocal laser scanning microscope, respectively.

### 2.9. Immunoblot Analysis

Total protein extracts were separated on 10–15% sodium dodecyl sulfate (SDS)-polyacrylamide gels and transferred to nitrocellulose membranes. The membranes were incubated with specific primary antibodies overnight at 4°C, and the immunoreactive antigen was recognized using HRP-conjugated secondary antibodies and an enhanced chemiluminescence detection kit (GE Healthcare, Buckinghamshire, UK).

### 2.10. Animals

All animal experiments were reviewed and approved by the Kyungpook National University Institutional Animal Care and Use Committee. Experiments were performed using 8-week-old male C57BL/6 mice with different genotypes, including wild-type (WT) *idh2*^+/+^ and knockout *idh2^−/−^* mice generated by breeding and identified by PCR genotyping, as previously described [27]. The mice were housed in microisolator rodent cages at 22°C with a 12 h light/dark cycle and allowed free access to water and standard mouse chow. Mice were divided into five groups, with 6–10 mice per group (WT, WT + acrolein, KO, KO + acrolein, and KO + acrolein + NAC). Mice were subjected to acute acrolein inhalation (10 ppm for 12 h), where NAC was intraperitoneally administered (500 mg/kg) 2 h before acrolein exposure.

### 2.11. Histological Analysis

For histological analysis, the lung tissues were isolated from mice after acrolein treatment and fixed in 4% formalin. Paraffin lung sections (5 *μ*m) were stained with hematoxylin and eosin (H&E) stain. Slides containing the lung sections were stained sequentially with hematoxylin gill number 3, bluing solution, and eosin Y by gently shaking at room temperature. To determine the airspace enlargement in the lungs [28], airspace areas were measured using ImageJ software. Lung injury was graded from 0 (normal) to 4 (severe) in four categories: interstitial inflammation, neutrophil infiltration, congestion, and edema [29]. Lung-injury score was calculated by adding the individual scores for each category. Grading was performed by an observer unaware of the treatment groups.

### 2.12. Terminal Deoxynucleotidyl Transferase-Mediated dUTP Nick-End Labeling (TUNEL) Staining

To evaluate apoptosis, the lung tissue sections were used for TUNEL staining using the In Situ Cell Death Detection Kit (Roche, Basel, Switzerland), according to the manufacturer's recommended protocol. TUNEL-stained slides were lightly counterstained with 4′,6-diamidino-2-phenylindole (DAPI) before final mounting. The stained slides were analyzed under an Axiovert 40 CFL microscope (Carl Zeiss AG; Oberkochen, Germany).

### 2.13. Statistical Analysis

Results are shown as the mean ± SD. Analyses were performed using a two-tailed *t*-test. *p* values *<* 0.05 were considered statistically significant.

## 3. Results and Discussion

### 3.1. Knockdown of *idh2* Exacerbates Cellular Apoptosis in Acrolein Insult

**Figure 1:**
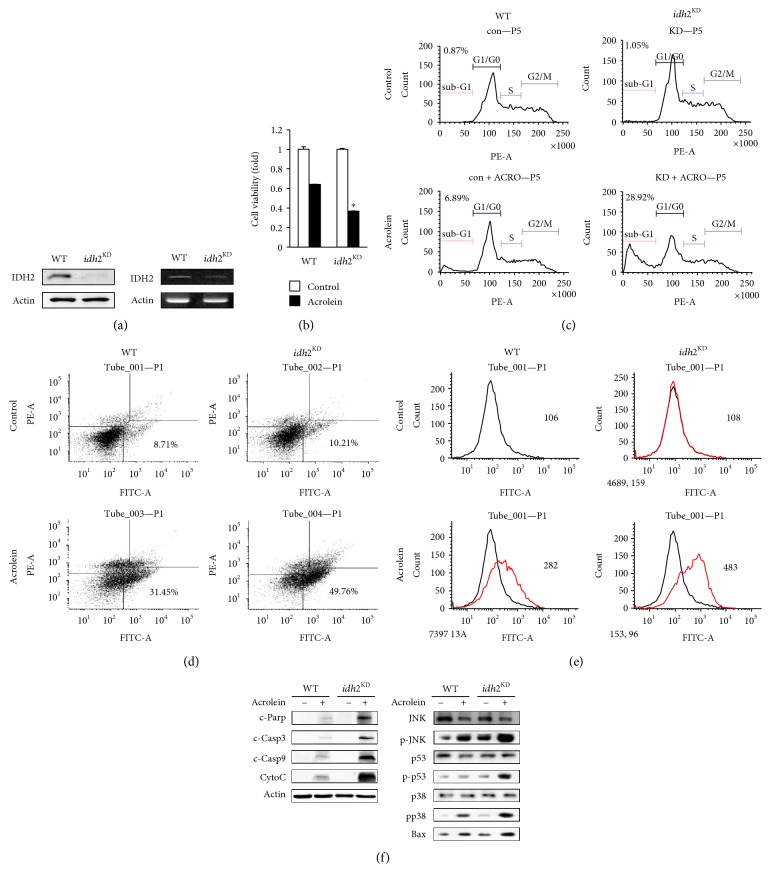
*Idh2* knockdown in LLC cells and their vulnerability to acrolein. (a) *idh2* protein expression levels were measured by immunoblotting using anti-*idh2* antibody. RT-PCR analysis of gene expression in WT and *idh2* knockdown (*idh2*^KD^) LLC cells. *β*-Actin was used as an internal control for the experiment. (b) Viability of control and *idh2*^KD^ LLC cells. Cells were cultured for 2 days at 37°C, exposed to 25 *μ*M acrolein for 1 h, and cell viability was then evaluated using MTT assay. Data are presented as the mean ± SD of four independent experiments. ^∗^*p* < 0.05 versus WT cells exposed to acrolein. (c) The ratio of cells undergoing apoptosis was measured by FACS. (d) Apoptosis was measured with FITC-labeled annexin in conjunction with PI. Cells were analyzed by flow cytometry. The lower right quadrants represent apoptotic cells. (e) Evaluation of apoptosis with annexin V by FACS. (f) Immunoblot analysis of apoptosis-related proteins. Control and *idh2*^KD^ cells were exposed to 25 *μ*M acrolein for 1 h. Cell extracts were electrophoresed on 10–15% SDS-polyacrylamide gels, transferred to nitrocellulose membranes, and immunoblotted with antibodies against cleaved caspase-3 (c-Casp3), cleaved caspase-9 (c-Casp9), cleaved PARP (c-PARP1), cytochrome c, p53, p-p53, p38, p-p38, JNK, p-JNK, and BAX. *β*-Actin was used as an internal control.

To investigate the role of IDH2 in acrolein-induced toxicity, we silenced the expression of *idh2* with shRNA. LLC cells were transfected with shRNA-encoding LVs targeting the transcript of murine *idh2* and were assayed to endogenously generate small RNA that mediates silencing of *idh2*. RT-PCR analysis revealed a significant decrease in *idh2* mRNA levels in *idh2* shRNA-transfected cells compared with nontarget shRNA-transfected cells, and immunoblot analysis revealed reduction in IDH2 protein expression levels in vector-infected cells (Figure 1(a)). To examine the effect of *idh2* knockdown on cell survival following acrolein treatment, LLC cells were treated with 25 *μ*M acrolein for 1 h. Acrolein treatment led to a significant decline in the viability of nontarget shRNA-transfected cells (Figure 1(b)). The effects of *idh2* knockdown on the cellular features of apoptosis were also examined.  Figure 1(c) shows a typical cell cycle plot of LLC cells that were transfected with control or *idh2* shRNA. The number of apoptotic cells was estimated by calculating the number of subdiploid cells in the cell cycle histogram. The number of apoptotic cells was markedly increased among the *idh2* shRNA-transfected cells compared to the control cells upon exposure to acrolein. To determine whether apoptosis is responsible for the vulnerability of *idh2*-silenced LLC cells to acrolein-induced cell damage, apoptotic cells were quantified using flow cytometry and PI/annexin V dual staining. The quantitative results in Figure 1(d) show that most of the acrolein-treated cells were in the early stage of apoptosis (depicted in the lower right quadrants). Similar results were also observed using the Annexin-V-FLUOS staining kit (Roche) with fluorescence-activated cell sorting (FACS) (Figure 1(e)). *Idh2*-silenced LLC cells were more vulnerable to acrolein-induced apoptotic cell death than their WT counterparts. The effect of *idh2* knockdown on the modulation of apoptotic marker proteins was also examined in LLC cells. As shown in Figure 1(f), caspase-3 and caspase-9 cleavages were more pronounced in the *idh2* shRNA-transfected cells. The formation of fragments indicative of proteolytic PARP cleavage, a proapoptotic marker, was significantly increased in *idh2* shRNA-transfected cells compared to control cells. In order to determine the role of IDH2 in acrolein-induced toxicity in the mitochondrial apoptotic pathway, the expression level of cytochrome c was evaluated. Upon exposure to acrolein, the level of cytochrome c was markedly increased in cells that were transfected with *idh2* shRNA (Figure 1(f)). The levels of proapoptotic proteins such as BAX were also significantly increased in *idh2* shRNA-transfected cells compared to control cells. To further evaluate the effect of *idh2* downregulation on the proapoptotic signaling pathway, the activation of p53 and JNK was examined by immunoblot analysis. The levels of phospho-p53 and phospho-JNK increased in cells that were transfected with *idh2* shRNA and treated with acrolein (Figure 1(f)). Activation of p38 has been implicated in the induction of apoptosis [30]. As shown in Figure 1(f), the level of phospho-p38 increased in LLC cells transfected with *idh2* shRNA upon exposure to acrolein. These findings reveal for the first time that IDH2 plays a vital role in LLC cell function and survival against acrolein toxicity.

### 3.2. Modulation of Redox Status by *idh2* Knockdown in Acrolein Toxicity

**Figure 2:**
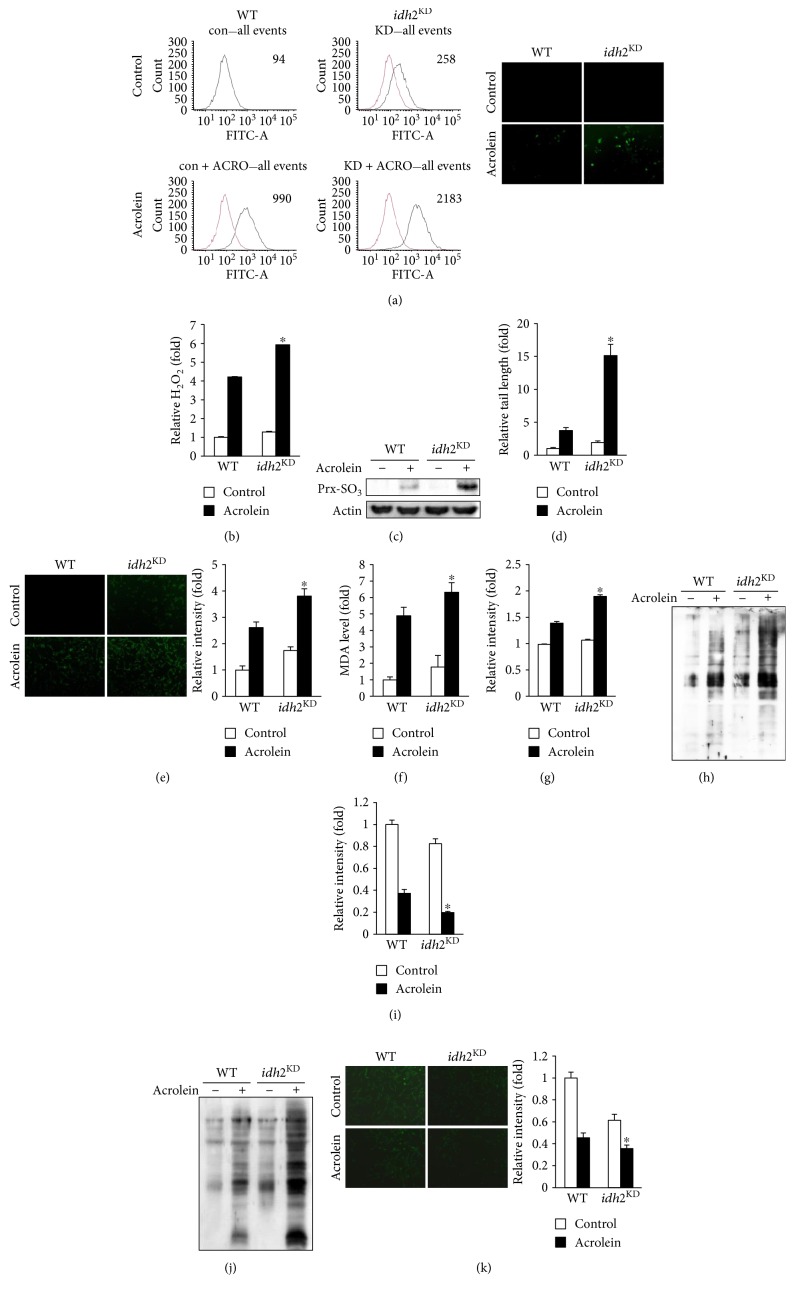
Effect of *idh2* knockdown on cellular redox status and oxidative damage in LLC cells exposed to acrolein. (a) LLC cells were stained with DCFH-DA for 30 min and DCF fluorescence was measured by flow cytometry and fluorescence microscopy. (b) Hydrogen peroxide production in LLC cells was measured by xylenol orange. (c) Immunoblot analysis of Prx-SO_3_ levels in LLC cell lysates. (d) Comet assay analysis of LLC cells and the tail/core width ratio obtained from captured images. (e) The levels of 8-OH-dG were measured by fluorescence microscopy. The average fluorescence intensity was calculated as previously described [44]. (f) The level of MDA accumulated in LLC cell extracts was determined using TBARS assay. (g) Cellular lipid peroxidation was measured with DPPP. The average fluorescence intensity was calculated as previously described [44]. (h) The level of oxidized protein adducts in the lung tissue extracts was measured with an anti-DNP antibody. (i) Fluorescence images of CMFDA-loaded cells were obtained under a microscope to evaluate cellular GSH levels. The average fluorescence intensity was calculated as previously described [44]. (j) The level of glutathionylated protein adducts in the lung tissue extracts was measured with an anti-GSH antibody. (k) Cellular NADPH level was determined by immunofluorescence using an anti-NADPH antibody. The average fluorescence intensity was calculated as previously described [44]. In (b), (d–g), (i), and (k), data are presented as the mean ± SD of four independent experiments. ^∗^*p* < 0.05 versus WT cells exposed to acrolein.

Excessive ROS are detrimental because they cause nonspecific oxidative damage to cellular components, DNA, proteins, lipids, and other macromolecules [31, 32]. In addition, ROS modulate redox homeostasis and redox-regulated signaling cascades, thereby causing further damage to tissues and cellular compartments [33]. To determine whether differences in susceptibility to acrolein toxicity between control and *idh2* shRNA-transfected cells were associated with ROS formation, the levels of intracellular peroxides in the cells were measured by FACS using the oxidant-sensitive probe, DCFH-DA. As shown in Figure 2(a), markedly increased ROS levels were observed in *idh2* shRNA-transfected cells exposed to acrolein. Furthermore, *idh2* knockdown was accompanied by a substantial elevation in intracellular H_2_O_2_ level, as measured by xylenol orange when cells were exposed to acrolein (Figure 2(b)). Increased levels of Prx-SO_3_, a marker for oxidative damage of the antioxidant enzyme Prx [34], were also found in *idh2* shRNA-transfected cells exposed to acrolein in comparison with control cells (Figure 2(c)). The occurrence of oxidative DNA damage, lipid peroxidation, and protein oxidation was evaluated as markers indicative of cellular oxidative damage. Next, the overall DNA fragmentation was measured using the Comet assay to evaluate DNA strand breaks induced by oxidative stress. As shown in Figure 2(d), the induction of DNA damage following acrolein treatment was augmented by knockdown of *idh2*. In this assay, damaged DNA exhibits the shape of a comet in which the tail length relates to the number of DNA strand breaks. To further confirm DNA damage, the level of 8-OH-dG, an indicator of oxidative DNA damage both in vivo and in vitro [25], was determined. After acrolein treatment, the endogenous DNA levels of 8-OH-dG were significantly increased in *idh2* shRNA-transfected cells as compared to control cells (Figure 2(e)). Consistent with the elevation of ROS, there was a considerable increase in the level of malondialdehyde (MDA), a lipid peroxidation marker, in *idh2* knockdown cells exposed to acrolein (Figure 2(f)). It has been shown that DPPP is a suitable probe for monitoring lipid peroxidation, specifically within the cell membrane [24]. Upon exposure to acrolein, DPPP fluorescence intensity was markedly increased in *idh2* shRNA-transfected cells in comparison with control cells (Figure 2(g)). To determine whether *idh2* knockdown increased sensitivity to protein damage, carbonyl contents were measured to evaluate protein oxidation. The carbonyl content of *idh2* shRNA-transfected cells was significantly higher than that of control cells (Figure 2(h)). An alternative method for monitoring oxidative stress within cells involves measuring the cellular levels of GSH, which is closely associated with many effects of acrolein on cell death [35, 36], using the GSH-sensitive fluorescent dye CFMDA [37]. Using this technique, GSH levels in *idh2* shRNA-transfected cells exposed to acrolein were found to be significantly decreased compared to that in control cells (Figure 2(i)). There are numerous oxidative stress-induced conditions during which the redox status and GSH/GSSG ratio are perturbed [38]. Protein S-glutathionylation is a posttranslational modification of protein sulfhydryl groups that occurs under oxidative stress [39]. The redox status of *idh2* shRNA-transfected cells was impaired more than that of control cells, as reflected by an increase in glutathionylated proteins (Figure 2(j)). NADPH, required for GSH generation by glutathione reductase, is an essential factor for cellular defense against oxidative damage. As expected, knockdown of *idh2* in LLC cells significantly decreased NADPH levels, which were further decreased upon exposure to acrolein (Figure 2(k)). Taken together, these results indicate that combination of *idh2* knockdown and acrolein exposure markedly elevated ROS generation and subsequently induced oxidative damage in LLC cells.

### 3.3. Role of *idh2* in the Mitochondrial Status Induced by Acrolein

**Figure 3:**
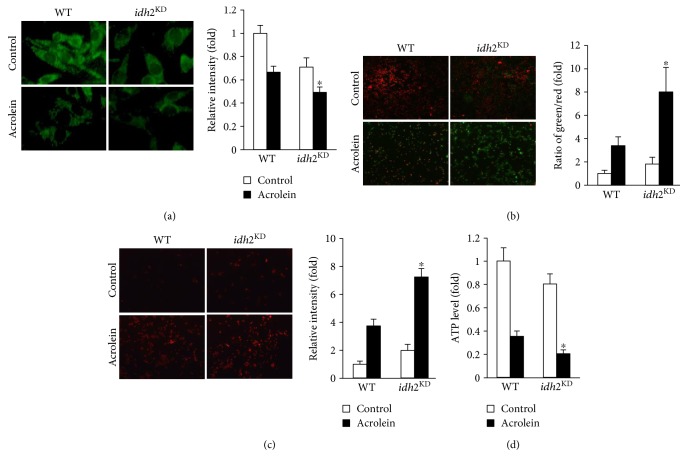
Downregulation of *idh2* aggravates mitochondrial dysfunction in LLC cells exposed to acrolein. (a) Mitochondrial membrane potential of LLC cells was measured by incorporation of Rh-123 dye into the mitochondria. (b) Membrane potential was analyzed with JC-1 probe. The mean (green/red) fluorescence intensity was expressed as a percentage compared with control. (c) MitoSox was used to detect mitochondrial ROS generation. MitoSox fluorescence was visualized by a fluorescence microscope. In (a–c), the average fluorescence intensity was calculated as previously described [44]. Data are presented as the mean ± SD of four independent experiments. ^∗^*p* < 0.05 versus WT cells exposed to acrolein. (d) ATP levels were measured in LLC cells following acrolein treatment. Data are presented as the mean ± SD of four independent experiments. ^∗^*p* < 0.05 versus WT cells exposed to acrolein.

In addition to the essential role of the mitochondria in energy metabolism, regulation of cell death, which presumably is associated with mitochondrial ROS production, has emerged as another major function of these organelles [40]. ROS play a major role in modulation of MPT, an important event in apoptosis [41]. The lipophilic cationic dye Rh-123 was used to determine changes in MPT in LLC cells exposed to acrolein. Acrolein-induced alteration of MPT, which was reflected by a decrease in Rh-123 fluorescence, was greater in *idh2* knockdown cells compared with control cells (Figure 3(a)). JC-1 is a fluorescent dye exhibiting potential-dependent accumulation in the mitochondria, which is commonly employed to detect MPT changes [26]. The ratio of green/red fluorescence also demonstrated that treatment of LLC cells with acrolein resulted in decreased MPT, and this effect was exacerbated by knockdown of *idh2* expression (Figure 3(b)). To assess whether changes in MPT were accompanied by alterations in intracellular ROS concentrations, the levels of intracellular peroxides in the mitochondria were evaluated using confocal microscopy and the mitochondrial oxidant-sensitive probe MitoSox. As shown in Figure 3(c), following *idh2* silencing, there was a marked increase in MitoSox fluorescence intensity after treatment of *idh2* knockdown cells with acrolein. In control cells, this increase in fluorescence intensity was less pronounced, indicating that *idh2* deficiency promotes production of mitochondrial ROS in acrolein-treated cells. The mitochondrial dysfunction in energy metabolism was determined by measuring ATP synthesis [42]. After exposure to acrolein, a significant decrease in ATP production was observed in *idh2* shRNA-transfected cells compared with control cells (Figure 3(d)). Taken together, these results indicate that *idh2* knockdown is associated with disruption of the mitochondrial redox environment and induction of mitochondrial dysfunction when cells are exposed to acrolein.

### 3.4. *Idh2* Deficiency Promotes Acrolein Toxicity In Vivo

**Figure 4:**
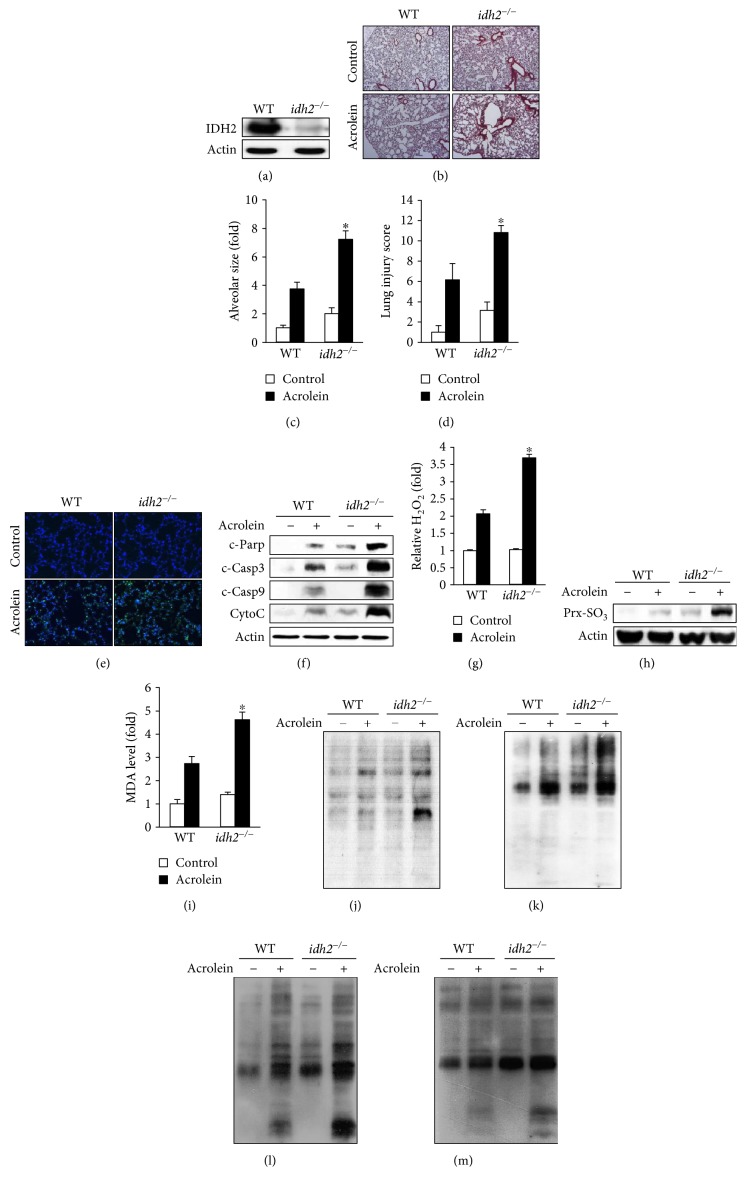
Acrolein-induced lung damage in *idh2^−/−^* mice. Mice were exposed to filtered air (control) or acrolein (10 ppm, 12 h). (a) Immunoblot analysis of IDH2 protein expression using an anti-IDH2 antibody. *β*-Actin was used as an internal control. (b) H&E-stained sections of the lung tissues after acrolein exposure. (c) Emphysema in acrolein-treated lung tissues assessed by mean alveolar airspace area (*μ*m^2^). (d) Lung injury scores were evaluated after acrolein exposure. (e) TUNEL staining of the lung tissues from acrolein-treated *idh2^−/−^* and WT (*idh2*^+/+^) mice. (f) Immunoblots comparing the levels of apoptotic marker proteins in the lung tissue extracts from acrolein-treated *idh2^−/−^* and WT mice. *β*-Actin was used as an internal control. (g) Intracellular H_2_O_2_ was measured using xylenol orange. (h) Immunoblot analysis of Prx-SO_3_ levels in the lung tissue extracts from WT and *idh2^−/−^* mice. (i) The levels of MDA accumulated in the lung tissue extracts were determined using TBARS assay. (j) The levels of acrolein-adducted proteins in the lung tissue extracts were measured with anti-acrolein antibody. (k) The levels of oxidized protein adducts, (l) glutathionylated protein adducts, and (m) nitrosylated protein adducts in the lung tissue extracts were measured with anti-DNP, anti-GSH, and anti-nitrotyrosine antibodies, respectively. In (c), (d), (g), and (i), data are shown as the mean ± SD (*n* = 3–6 mice in each group). ^∗^*p* < 0.05 versus acrolein-treated WT mice.

To test the physiological relevance of *idh2* deficiency in acrolein toxicity, WT (*idh2*^+/+^) mice and mice lacking *idh2* (*idh2^−/−^*) were exposed to 10 ppm acrolein for 2 h or filtered air (control). As expected, protein expression of IDH2 was not detected in the lung tissues harvested from *idh2^−/−^* mice (Figure 4(a)). Histological analysis of the lung tissue showed that acrolein caused enlargement of alveolar and immune cell infiltration, and these histological characteristics were dramatically enhanced in *idh2^−/−^* mice compared with *idh2*^+/+^ mice (Figure 4(b)). The significant increase in susceptibility of *idh2^−/−^* mice to acrolein-induced lung injury was also reflected by a significant increase in alveolar airspace area (Figure 4(c)) and lung injury score (Figure 4(d)). To gain further insights into the effect of IDH2 on lung damage in acrolein-treated mice, the effect of *idh2* deficiency on the process of apoptosis was studied. As shown in Figure 4(e), more TUNEL-stained spots were observed in the lung tissues of *idh2^−/−^* mice compared with WT mice. Western blot analysis indicated that cleaved caspase-3, cleaved caspase-9, and cleaved PARP, which represent apoptotic index, were increased in the lung tissue of *idh2^−/−^* mice exposed to acrolein as compared to control. Increased expression of cytochrome c, representative of mitochondrial apoptosis, was also identified in the lung tissue of *idh2^−/−^* mice upon acrolein exposure (Figure 4(f)). These results suggest that the difference in severity of acrolein-induced lung damage depends on the presence of *idh2*, which influences the level of apoptosis. The major enzyme to generate mitochondrial NADPH is the mitochondrial isoenzyme, IDH2 [21]. Thus, deficiency of *idh2* may induce an imbalance of redox status in the mitochondria, subsequently increasing the vulnerability of lung cells to acrolein. To determine whether the differences in acrolein-induced lung cell death observed between WT and *idh2^−/−^* mice were associated with ROS formation, the levels of intracellular hydrogen peroxide in the lung tissue were measured using xylenol orange. As depicted in Figure 4(g), a significantly higher level of intercellular hydrogen peroxide was observed in the lung tissue of acrolein-treated *idh*2*^−/−^* mice compared to that in WT mice. Increased expression levels of Prx-SO_3_, a marker of oxidative stress, were also observed in the lung tissue of acrolein-treated *idh*2*^−/−^* mice (Figure 4(h)). Additionally, the levels of MDA (an indicator of lipid peroxidation) and acrolein-adducted proteins, and the level of oxidative protein damage that was measured by determining the number of derivatized carbonyl groups on oxidized proteins by immunoblotting, were significantly higher in the lung tissues of acrolein-treated *idh*2*^−/−^* mice compared to those in control mice (Figures 4(i) and 4(k)). On exposure to acrolein, the redox status of *idh*2*^−/−^* mice was impaired more than that of *idh*2^+/+^ mice, as reflected by an increase in glutathionylated proteins in the lung tissues (Figure 4(l)). In addition, nitrotyrosine immunoreactivity was found to be relatively stronger in the lung tissue of acrolein-treated *idh2^−/−^* mice compared with that in *idh2*^+/+^ mice, indirectly reflecting the higher level of reactive nitrogen species in *idh2^−/−^* mice (Figure 4(m)). Collectively, these results supported the notion that *idh2* deficiency deteriorates the mitochondrial redox status that aggravates acrolein-induced colitis through apoptosis.

### 3.5. Protective Effects of NAC against Acrolein Toxicity In Vitro and In Vivo

**Figure 5:**
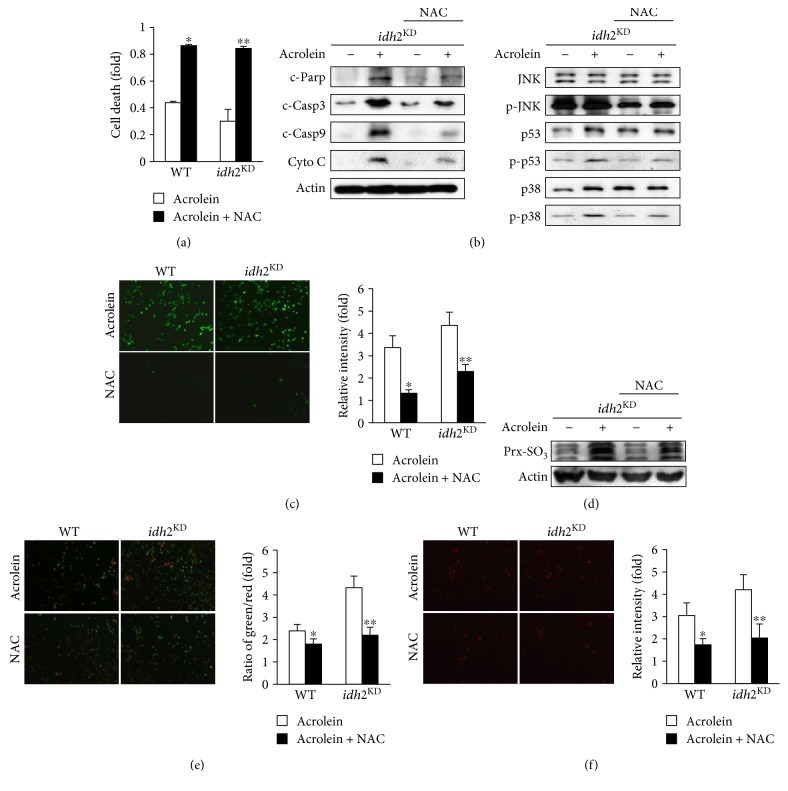
The effect of NAC on acrolein-induced toxicity in *idh2* knockdown LLC cells. (a) Viability of acrolein-exposed *idh2*^KD^ LLC cells in the presence and absence of 1 mM NAC for 2 h was evaluated using MTT assay. Data are presented as the mean ± SD of four independent experiments. ^∗^*p* < 0.05 versus control cells exposed to acrolein. (b) Immunoblot analysis of apoptosis-associated proteins in *idh2*^KD^ LLC cells. *β*-Actin was used as an internal control. (c) Intracellular ROS levels were measured with DCFH-DA and fluorescence microscopy. (d) Immunoblot analysis of Prx-SO_3_ levels in *idh2*^KD^ LLC cell lysates. (e) Mitochondrial membrane potential of *idh2*^KD^ LLC cells was measured by incorporation of Rh-123 dye into the mitochondria. (f) MitoSox was used to detect mitochondrial ROS generation in *idh2^KD^* LLC cells. MitoSox fluorescence was visualized by a fluorescence microscope. In (c), (e), and (f), the average fluorescence intensity was calculated as previously described [44]. Data are presented as the mean ± SD of four independent experiments. ^∗^*p* < 0.05 versus WT cells and ^∗∗^*p* < 0.05, versus *idh2*^KD^ cells exposed to acrolein.

**Figure 6:**
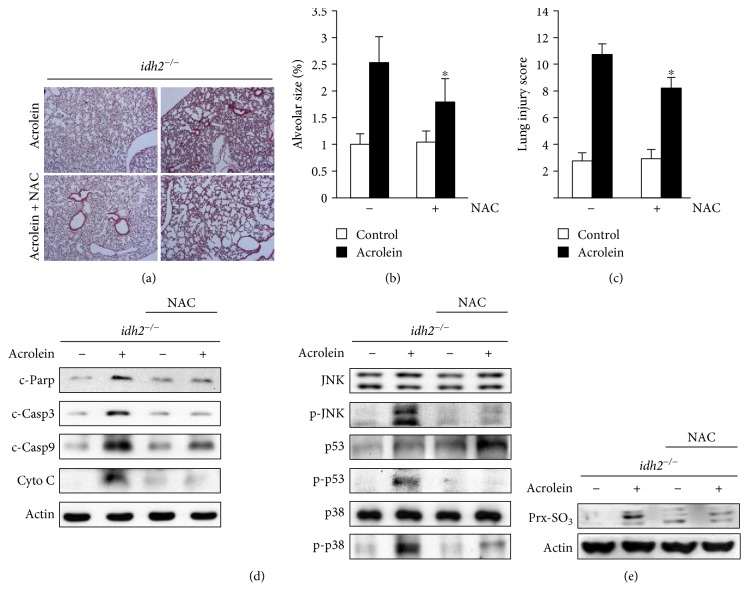
Protective effects of NAC against acrolein-induced lung damage in *idh2^−/−^* mice. NAC (500 mg/kg) was intraperitoneally administered to *idh2^−/−^* mice 2 h prior acrolein exposure. (a) H&E-stained sections of the lung tissue after acrolein administration. (b) Emphysema in acrolein-treated lung tissues assessed by mean alveolar airspace area (*μ*m^2^). (c) Lung injury scores were evaluated after acrolein exposure. (d) Immunoblots comparing the levels of apoptotic marker proteins in the lung tissue extracts from *idh2^−/−^* mice. *β*-Actin was used as an internal control. (e) Immunoblot analysis of Prx-SO_3_ levels in the lung tissue extract from *idh2^−/−^* mice. In (b) and (c), data are shown as the mean ± SD (*n* = 3–6 mice in each group). ^∗^*p* < 0.05 versus acrolein-treated and NAC-untreated *idh2^−/−^* mice.

To confirm the influence of increased oxidative stress on acrolein-induced damage, the effect of the thiol antioxidant NAC on acrolein toxicity was evaluated both *in vitro* and *in vivo*. It was previously shown that NAC reduced oxidative stress by improving the thiol redox status [43]. Pretreatment of *idh2* shRNA-transfected LLC cells with 1 mM NAC efficiently suppressed cell viability loss (Figure 5(a)) and apoptotic cell death (Figure 5(b)) after exposure to acrolein. Cellular oxidative stress, reflected by an increase in DCF fluorescence (Figure 5(c)) and Prx-SO_3_ level (Figure 5(d)), was significantly attenuated in acrolein-treated *idh2* knockdown cells pretreated with NAC. Pretreatment of *idh2* knockdown cells with NAC significantly inhibited acrolein-induced disruption of MPT, reflected by JC-1 fluorescence ratios (Figure 5(f)) and increased mitochondrial ROS levels as evaluated by MitoSox (Figure 5(f)). To investigate the protective effects of NAC on acrolein-induced lung damage in vivo, NAC (500 mg/kg) was intraperitoneally administered to mice 2 h prior to acrolein exposure. The histological characteristics of acrolein-administered *idh2^−/−^* mice pretreated with NAC were compared with those of *idh2^−/−^* mice treated with acrolein alone. Compared with acrolein-administered *idh2^−/−^* mice pretreated with NAC, *idh2^−/−^* mice treated with acrolein alone showed an increased susceptibility to lung injury, as reflected by their histological characteristics (Figure 6(a)), airspace area (Figure 6(b)), and lung injury score (Figure 6(c)). In relation to apoptosis, the level of apoptotic marker proteins was evaluated in the lung tissue. The levels of the cleaved form of caspase-3, caspase-9, PARP, and cytochrome c and the activated forms of p38, p53, and JNK in acrolein-administered NAC-pretreated *idh2^−/−^* mice were attenuated compared to those in *idh2^−/−^* mice treated with acrolein alone (Figure 6(d)). Upon exposure to acrolein, the level of Prx-SO_3_ was significantly lower in NAC-pretreated mice compared to untreated mice (Figure 6(e)). These results suggest that acrolein toxicity in vitro and in vivo is ameliorated by NAC through protection against oxidative stress that results from *idh2* deficiency.

## 4. Conclusion

The present study demonstrates that *idh2* deficiency leads to increased susceptibility to acrolein-induced toxicity in LLC cells and lung tissue in mice through disruption of the mitochondrial redox status. Having established the importance of IDH2 in the regulation of redox status and in lung tissue functions, we hereby propose that downregulation of *idh2* in both in vitro and in vivo systems could be an effective model for future lung disease research. Furthermore, our study provides a useful model to investigate the potential application of NAC in the treatment or prevention of acrolein toxicity.
